# Perioperative Risk in Adults with Congenital Heart Disease Undergoing Non-Cardiac Surgery: Challenges and Tailored Strategies

**DOI:** 10.3390/jcm14103340

**Published:** 2025-05-11

**Authors:** Magalie Ladouceur, Lena Valacco, Zied Ltaief, Tobias Rutz, Sébastien Hascoet, Judith Bouchardy

**Affiliations:** 1 Service de Cardiologie, University Hospitals of Geneva, 1205 Geneva, Switzerland; judith.bouchardy@chuv.ch; 2 Service de Cardiologie, Centre Hospitalier Universitaire Vaudois, Université de Lausanne, 1011 Lausanne, Switzerland; lena.valacco@unil.ch (L.V.); tobias.rutz@chuv.ch (T.R.); 3 Department of Pediatric Cardiology and Congenital Heart Disease, Marie Lannelongue Hospital, Groupe Hospitalier Paris Saint Joseph, 92350 Le Plessis-Robinson, France; 4 Department of Adult Intensive Care Medecine, Centre Hospitalier Universitaire Vaudois, Université de Lausanne, 1011 Lausanne, Switzerland; zied.ltaief@chuv.ch

**Keywords:** non-cardiac surgery, anesthesiology, congenital heart disease, risk stratification

## Abstract

Advances in surgical and medical management of congenital heart disease have improved survival rates, leading to a growing population of adult congenital heart disease (ACHD) patients requiring specialized perioperative care. Studies indicate that ACHD patients undergoing non-cardiac surgery (NC surgery) have increased mortality and morbidity risks compared to the general population, with complication rates particularly high in those with complex defects, such as Fontan circulation, Eisenmenger syndrome, or cyanotic congenital heart disease. Key perioperative concerns include hemodynamic instability, arrhythmias, thromboembolic events, and bleeding risks. Additionally, comorbidities, such as frailty, chronic inflammation, or respiratory disease, further complicate perioperative management. Multidisciplinary collaboration is critical, involving cardiologists, anesthesiologists, and surgeons to optimize preoperative preparation and perioperative monitoring. Preoperative risk stratification is essential, integrating congenital heart lesion complexity, functional status, and procedural risk. This review underscores the importance of structured preoperative assessment, appropriate risk evaluation, and individualized perioperative strategies to improve surgical outcomes in ACHD patients undergoing NC surgery. Further research is needed to refine risk prediction models and optimize perioperative protocols tailored to this unique patient population.

## 1. Epidemiology

Congenital heart disease (CHD) is one of the most frequently occurring congenital anomalies, affecting approximately 0.8% of live births. Over the past decades, advancements in medical and surgical interventions have led to a dramatic improvement in survival rates, particularly for those requiring neonatal corrective procedures. The ability to successfully manage CHD in early life has significantly altered the long-term prognosis of affected individuals. Notably, the survival rate following corrective cardiac surgery has reached approximately 97%, enabling most children with CHD to transition into adulthood [1]. The extent of survival, however, varies based on the severity of the congenital anomaly. Data indicate that nearly 98% of individuals with mild CHD, 90% of those with moderate defects, and 56% of those with complex congenital anomalies now survive beyond 18 years of age [2]. Improved survival has resulted in a steady expansion of the ACHD population, with prevalence increasing from 1.47 per thousand adults in 2010 to 1.83 in 2025, and an estimated 2.31 per thousand by 2050. This represents an average annual growth rate of approximately 1.5% between 2010 and 2025, gradually slowing to about 1.1% per year by 2050 as the adult CHD population begins to plateau [3].

With this demographic shift, challenges emerge, including the need for specialized perioperative care when ACHD patients undergo non-cardiac surgery (NC surgery). According to a study utilizing the Nationwide Inpatient Sample database (2002–2009), ACHD patients undergoing non-cardiac surgery represented an increasing proportion of all non-cardiac surgical admissions over time, with an increase from 0.07% in 2002 to 0.18% in 2009 [4]. Similarly, an analysis of the 2010–2018 Nationwide Readmissions Database found that the prevalence of ACHD in major elective surgeries increased from 0.06% to 0.17%, reflecting a 183% increase over the study period [5].

ACHD patients undergo a variety of non-cardiac procedures, with gastrointestinal, orthopedic, and thoracic surgeries being the most frequently performed [4,5] (Table 1).

Despite increased survival rates into adulthood, ACHD patients face higher perioperative risks than their non-ACHD counterparts [6]. Indeed, inpatient mortality was significantly higher among ACHD patients (4.1%) compared to non-ACHD patients (3.6%). After adjusting for confounders, ACHD was identified as an independent predictor of increased perioperative mortality, regardless of the type of surgery [4]. Williamson et al. reported similar findings in their analysis of major elective non-cardiac surgeries [5]. They conducted a retrospective cohort study using the U.S. Nationwide Readmissions Database from 2010 to 2018, identifying over 4.9 million adults who underwent major elective surgeries, among whom 0.11% had a prior diagnosis of CHD. After risk adjustment, ACHD patients had a 76% increased risk of in-hospital mortality compared to non-ACHD (adjusted risk ratio: 1.76, 95% CI 1.25 to 2.47). In addition to increased mortality, ACHD patients experience higher rates of perioperative morbidity. Maxwell et al. found that the composite morbidity endpoint (including acute kidney injury, pneumonia, respiratory failure, stroke, myocardial infarction, and thromboembolism) was significantly more common in ACHD patients (21.4% vs. 16.0% in non-ACHD patients) [4]. Similarly, Williamson et al. reported an increased risk of in-hospital complications, prolonged hospital stays, and higher healthcare costs [5]. Beyond in-hospital outcomes, ACHD patients also face a higher risk of postoperative complications and readmission. ACHD patients had a 32% increased risk of readmission within 30 days after major elective non-cardiac surgery [5]. These readmissions were often due to cardiovascular instability, arrhythmias, and pulmonary complications.

The severity of CHD plays a crucial role in determining perioperative risk. Patients with complex ACHD lesions had the highest mortality rate (7.3%) compared to those with simple lesions (3.8%) [4]. Likewise, complication rates increased with CHD severity, with patients having single-ventricle physiology suffering the highest risk (15% to 43.7% complication rate) [5,7].

This underscores the critical need for comprehensive preoperative risk stratification and timely referral of patients with complex CHD to specialized centers. Ensuring that elective surgeries are performed at high-volume institutions with expertise in CHD management allows for specialized perioperative care, ultimately improving patient safety and surgical outcomes [8].

## 2. Risk Stratification

The perioperative cardiovascular risk in patients undergoing NC surgery is determined by two major factors: patient-related risks and surgery-related risks. These two dimensions interact dynamically and contribute to the overall surgical risk. A proper risk assessment should take both elements into account to guide perioperative management.

### 2.1. Surgery-Related Risk

Several factors influence surgical risk, including the timing of the procedure, the surgical approach, the anesthetic modalities, the duration of the intervention, and the experience of the surgical center.

Surgery-related risk is determined by the intrinsic characteristics of the procedure, including its invasiveness, duration, urgency, and associated hemodynamic stress. The European Society of Cardiology guidelines classify surgical risk based on the expected 30-day cardiovascular mortality, myocardial infarction, and stroke rate [8].

Immediate procedures (e.g., ruptured aortic aneurysm repair) carry the highest risk due to the inability to optimize preoperative status, while urgent procedures (e.g., emergency appendectomy) have a moderate risk. Time-sensitive procedures (e.g., cancer surgery) require early intervention, though some preoperative optimization may be possible. Finally, elective procedures, where surgery can be scheduled at an optimal time, tend to have the lowest cardiovascular risk [8]

Certain types of surgeries are inherently more stressful on the cardiovascular system. The ESC classifies surgical risk as high-risk procedures (>5% cardiac event risk), intermediate-risk procedures (1–5% cardiac event risk), and low-risk procedures (<1% cardiac event risk; Table 2).

The surgical approach may increase hemodynamic stress. Laparoscopic surgery generally induces less hemodynamic stress than open surgery, while endovascular and minimally invasive techniques reduce perioperative risk compared to conventional open surgical approaches.

Varied outcomes have been observed in NC surgeries among ACHD. Williamson et al. studied major elective NC surgery in ACHD patients. They identified a higher risk of mortality in abdominal aortic aneurysm surgery, colectomy, and hepatectomy, as well as an increased risk of complications following esophagectomy and gastrectomy [5]. This elevated perioperative risk was further confirmed for thoracoabdominal cancer resections, as demonstrated by Sakowitz et al. [9].

Finally, general anesthesia is associated with more profound hemodynamic alterations compared to regional anesthesia, which may be preferable in high-risk cardiac patients.

Additional considerations should be kept in mind. Any surgical procedure can induce a systemic inflammatory response, activation of the sympathetic nervous system, and prothrombotic changes. Fluid shifts and blood loss can lead to hypotension or increased vascular resistance, potentially worsening the myocardial oxygen supply/demand balance. High-risk surgeries increase the likelihood of perioperative bleeding, which is especially concerning for patients on antiplatelet therapy or anticoagulants. Postoperative prothrombotic states may contribute to venous thromboembolism and cardiovascular complications.

### 2.2. Patient-Related Risk

Among the various factors influencing surgical outcomes, patient-related risk plays a crucial role in determining perioperative morbidity and mortality. Maxwell et al. analyzed 21 cases from the Anesthesia Closed Claims Project database, focusing on adverse events in ACHD patients undergoing surgery (*n* = 11) [10]. Their findings revealed that nearly half of the reported cases involved non-cardiac procedures (*n* = 10), with the type of congenital heart disease (50%), inadequate preoperative assessment or optimization (40%), and insufficient postoperative monitoring (50%) being the primary contributors to negative outcomes. Interestingly, surgical techniques themselves were seldom the direct cause of complications, reinforcing the need for ACHD expertise and the importance of comprehensive patient evaluation and perioperative planning.

Preoperative risk assessment serves as the foundation for informed decision-making, benefiting both physicians and patients. A structured evaluation helps tailor the surgical approach and perioperative monitoring strategy, ensuring that the level of risk is proportionate to the expected benefits. This process is particularly relevant for elective surgeries, where outcomes can be optimized through careful planning. In determining whether surgery is appropriate, the decision must balance the risk of operating versus the risk of withholding surgery. Unlike acute or life-threatening conditions where immediate intervention is required, elective procedures demand a broader assessment that accounts for long-term survival, quality of life, and functional prognosis.

Despite efforts to stratify perioperative risk, existing surgical risk models fail to account for the specific challenges of ACHD patients. Widely used tools, such as the Revised Cardiac Risk Index (RCRI) and the American College of Surgeons National Quality Improvement Program (ACS-NSQIP) Risk Model, primarily consider common cardiovascular comorbidities, such as heart failure, renal dysfunction, and respiratory failure. However, they do not integrate factors unique to congenital heart disease, such as lesion complexity, functional reserve, and long-term adaptations to abnormal circulation. The predictive accuracy of these models varies, with reported area under the curve values ranging from 0.68 to 0.92, yet none of these tools have been validated specifically in ACHD populations [8].

To refine perioperative risk prediction, additional markers may improve risk stratification beyond standard risk models. Functional assessments, such as peak VO_2_, and advanced cardiac imaging techniques provide valuable insight into the patient’s cardiovascular reserve. Hemodynamic parameters, electrophysiological data, and biomarkers, such as NT-proBNP and troponins, offer further refinement in risk estimation. Integrating these factors into clinical practice could lead to more precise perioperative risk models, ultimately improving surgical outcomes for ACHD patients.

Beyond individual risk assessment, multidisciplinary coordination is fundamental to achieving optimal outcomes. Collaboration between surgeons, cardiologists, anesthesiologists, and intensivists allows for a more refined evaluation of surgical feasibility. This interaction ensures that perioperative management strategies are tailored to the patient’s unique hemodynamic profile and residual cardiac lesions, reducing the likelihood of adverse outcomes. The involvement of the patient and their family in shared decision-making is equally critical, ensuring that expectations are clearly set, and that informed consent is based on a realistic understanding of the risks and benefits of surgery.

## 3. Risk in Specific CHD Lesions

**Figure 1:**
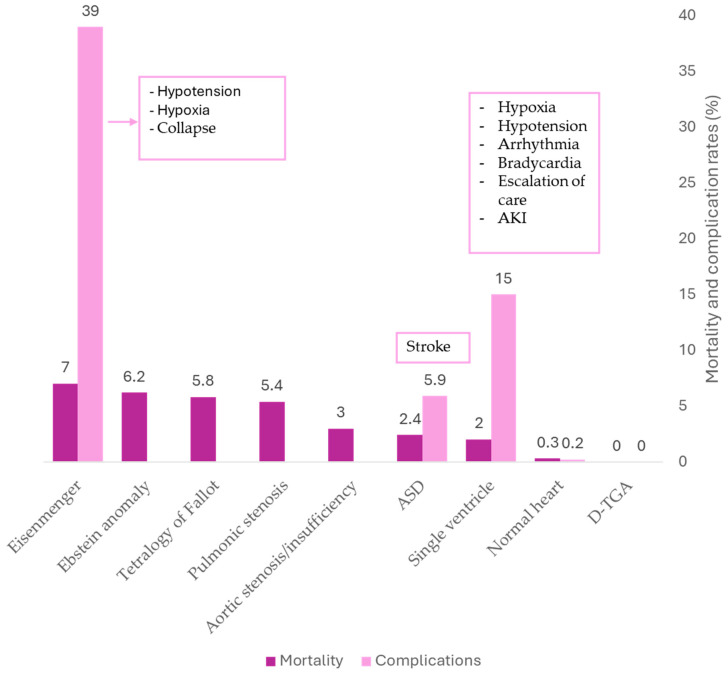
Mortality rates and complication rates according to congenital heart defect. Values were extracted from the studies of Bennett et al. [11], Maxwell et al. [4], Christenser et al. [12], and Villablanca et al. [13]. Complications are listed in boxes within the figure when available. They correspond to cardiac, renal, infectious, intraoperative, pulmonary, and neurological categories reported in patients with structurally normal hearts. Only the most frequent complications were listed for single-ventricle physiology (those accounting for more than 5% of all reported complications). Mortality corresponded to all-cause in-hospital mortality. ASD, atrial septal defect; AKI, acute kidney injury; D-TGA, dextro-transposition of the great arteries.

ACHD patients undergoing NC surgery face varying degrees of risk depending on the underlying cardiac defect, hemodynamic consequences, and associated complications. Several studies have identified increased perioperative complications in specific ACHD subgroups, including those with atrial septal defect (ASD), single-ventricle physiology, transposition of the great arteries, cyanotic congenital heart disease, and Eisenmenger syndrome. The interaction between these structural defects and surgical stressors contributes to higher perioperative morbidity and mortality (Figure 1).

### 3.1. Patients with ASD/PFO

Even if they are simple defects, ASD/PFO have a well-documented increased risk of postoperative ischemic stroke. A study analyzing over 19 million non-cardiac surgeries found that patients with a pre-existing ASD/PFO had a 5.9% risk of stroke after their operation, compared to 0.02% in patients without these defects—a nearly 17-fold increase in risk [13]. Furthermore, ASD/PFO patients had a 4-fold increase in in-hospital mortality and a 7-fold increase in stroke within 30 days of surgery. In another patient-based study that excluded patients without an echocardiogram during the surgical admission to ensure ASD/PFO diagnosis, Smilowitz et al. reported a 6-fold increased risk of perioperative stroke in patients with ASD or PFO [14]. The risk was particularly high in major NC operations, such as general, obstetric, gynecologic, orthopedic, and vascular surgeries, suggesting that surgery-induced hemodynamic fluctuations may exacerbate embolic mechanisms.

### 3.2. Patients with Fontan Circulation

The Fontan circulation represents a surgically palliated single-ventricle physiology, where systemic venous return bypasses the heart and passively flows into the pulmonary arteries without a sub-pulmonary pump. While this approach has allowed for survival into adulthood, Fontan patients remain high-risk candidates for NC surgery due to chronic venous congestion, low cardiac output, and multi-organ dysfunction. A large retrospective study analyzing 538 NC procedures in 154 Fontan patients reported an overall 15% complication rate. Complications were mostly intraoperative, occurring in 12% of procedures. The most common were hypoxia (7%), hypotension (3%), and arrhythmias (2%). Postoperative complications were less frequent, with escalation of the level of care being the most common (2%), followed by hypotension, acute kidney injury, and heart failure, each occurring in less than 1% of cases [7]. The risk was significantly higher in cyanotic Fontan patients (SpO_2_ < 90%), who had a three-fold increase in complications. Most complications occurred during surgery (12%), while 5% developed postoperatively. Mortality rates were also higher in patients requiring general anesthesia, reaching 2%.

The predominance of intraoperative complications is readily explained by the unique pathophysiology of the Fontan circulation, which is particularly vulnerable to hemodynamic instability during anesthesia and mechanical ventilation. Due to the passive nature of pulmonary blood flow, mechanical and positive-pressure ventilation can severely reduce cardiac output by increasing intrathoracic pressure and decreasing venous return. Additionally, anesthesiologists must carefully manage the acid–base balance, as both metabolic and respiratory acidosis can significantly elevate pulmonary vascular resistance, worsening hemodynamic instability. The use of pulmonary vasodilators, such as oxygen and nitric oxide, can help enhance pulmonary blood flow and support cardiac output during surgery, reducing the risk of perioperative complications [15]. The type of non-cardiac surgery significantly influences intraoperative outcomes in Fontan patients. High positive end-expiratory pressure or increased intra-abdominal pressure during laparoscopic surgery can decrease venous blood return and reduce cardiac output. However, case reports have shown that laparoscopic surgery can be performed with acceptable risk if intra-abdominal pressures are <10 mmHg in patients with Fontan physiology [16].

Major surgical procedures, particularly those involving fluid shifts or prolonged anesthesia, may be associated with higher complication rates and prolonged hospitalization. A study by Brown et al. found that major surgeries required intraoperative vasopressors in 39% of cases and had a 64% risk of extended hospitalization [17]. In contrast, minimally invasive procedures and short-duration surgeries had lower complication rates, and 36.3% of patients undergoing minor procedures were discharged on the same day. Key predictors of failed same-day discharge included undergoing a major surgical procedure, need for intraoperative vasopressors, and pre-existing organ dysfunction. Anesthetic agents, such as propofol and volatile anesthetics, can cause profound vasodilation and worsen hypotension, necessitating careful vasopressor management. Studies suggest that regional anesthesia, when feasible, may provide a safer alternative by preserving spontaneous ventilation [7].

Patients with a Fontan fenestration or the development of veno-venous collaterals inevitably experience a right-to-left shunt, leading to persistent cyanosis at rest. This circulatory alteration significantly heightens the risk of paradoxical embolism, necessitating the use of air filters to minimize embolic events. While central venous access and peripherally inserted central catheters can assist in hemodynamic monitoring and vascular access, their use in Fontan patients carries an increased risk of thrombosis due to altered venous flow dynamics and hypercoagulability associated with this physiology [18]. Finally, chronic cyanosis and associated liver disease may increase the bleeding risk (see sections below).

### 3.3. Patients with a Transposition of the Great Arteries

Patients with a systemic right ventricle (e.g., those post-Mustard or Senning procedures for D-transposition of the great arteries and patients with congenitally corrected transposition of the great arteries) exhibit an increased risk of heart failure, arrhythmias, and sudden death [19] that may be enhanced during NC surgery. Long-term strain on the right ventricle, pumping against systemic circulation, predisposes these patients to ventricular dysfunction. However, there is limited data on perioperative outcomes in patients with transposition of the great arteries. A retrospective analysis of patients with surgically corrected D-TGA undergoing NC surgery found that 10% experienced perioperative complications, including bradycardia, failed extubation, and significant bleeding reoperation [12]. However, most of the patients were children with an arterial switch operation.

### 3.4. Patients with Cyanotic Congenital Heart Disease and Eisenmenger

Patients with cyanotic congenital heart disease (CCHD) represent a highly diverse group, characterized by reversed or bidirectional shunting. They can generally be categorized into two main subgroups: (1) those with limited pulmonary blood flow and (2) those who develop pulmonary vascular disease due to an unrestricted connection between the systemic and pulmonary circulations. The central issue remains determining the presence or absence of pulmonary vascular disease and pulmonary arterial hypertension [20].

The combination of polycythemia, coagulopathy, and limited oxygen reserve significantly increases the likelihood of hypoxia, arrhythmias, thrombus and paradoxical embolism, and excessive bleeding during and after surgery.

For instance, cyanosis has been identified as a major predictor of perioperative complications, with studies showing a 20% complication rate, higher than in non-cyanotic Fontan patients [7]. Among the highest-risk ACHD patients are those with Eisenmenger syndrome, a condition defined by longstanding left-to-right shunting that results in pulmonary vascular obstructive disease and irreversible shunt reversal. Nowadays, many patients survive into their fifth decade, though symptoms worsen with age [21]. Managing anesthesia in Eisenmenger patients is particularly challenging, as even minor hemodynamic disturbances can precipitate severe hypoxia and cardiovascular collapse. Due to their fixed pulmonary vascular resistance, these patients rely on systemic vascular resistance (SVR) to maintain perfusion, and any decrease in SVR increases right-to-left shunting and worsens hypoxia. Most of the complications are intraoperative. A retrospective review of 33 Eisenmenger patients undergoing 53 non-cardiac surgeries reported a 26% incidence of hypotension, all occurring during induction of general anesthesia, with the rate rising to 39% among those receiving general anesthesia. Seventeen percent experienced oxygen desaturation, with more than half of these episodes preceded by hypotension [11]. Mortality rates ranged from 3.8% to 7%, with risk factors including inhalational anesthesia without vasopressor support, cyanosis, and significant hemodynamic instability [11,22]. When propofol was used without vasopressors, 83% of patients experienced profound hypotension [11]. However, nearly all anesthetic agents reduce SVR, exacerbating shunting, reinforcing the need for careful perioperative planning.

Secondary erythrocytosis is common due to chronic hypoxia-driven erythropoietin production, leading to increased blood viscosity and thrombotic risk [23]. Patients with Eisenmenger syndrome exhibit a paradoxical risk of both bleeding and thrombosis due to platelet dysfunction, coagulation factor deficiencies, and right-to-left shunting [24]. This predisposes them to spontaneous hemoptysis, gastrointestinal bleeding, and intracranial hemorrhage, while simultaneously increasing the risk of paradoxical embolism, particularly in atrial arrhythmia.

Preoperative optimization of fluid balance, coagulation status, and iron levels is essential to reducing surgical risk. For patients undergoing major procedures, the coagulation factor and platelet replacement should be considered to minimize bleeding complications. In cases of significant thrombocytopenia, controlled venesection may be performed prior to surgery to enhance platelet availability and improve hemostasis [25]. Some studies also propose preoperative isovolumic phlebotomy for patients with hematocrit levels exceeding ~65% to minimize intraoperative bleeding and reduce blood viscosity, with the extracted blood preserved for potential autotransfusion during surgery [15,26,27]. Addressing iron deficiency is also crucial in planned interventions to support overall recovery. During the perioperative period, maintaining adequate hydration with intravenous fluids is necessary to prevent hyperviscosity-related complications and ensure optimal circulation.

Fluid management is particularly challenging, as Eisenmenger physiology is preload-dependent. Blood loss should be replaced cautiously, avoiding both hypovolemia and volume overload, which could lead to right ventricular failure. Intraoperative monitoring is essential, but central venous access should be placed cautiously to avoid air embolism, which can paradoxically enter systemic circulation through the shunt. Air filters on all intravenous lines are recommended.

After surgery, intensive monitoring is crucial, as even minor hemodynamic fluctuations can cause decompensation. Effective pain control is also critical, as poorly managed pain can increase pulmonary valve resistance, SVR, and myocardial oxygen demand, worsening hemodynamic instability.

Finally, infective endocarditis is another significant concern, as abnormal vascular structures and turbulent flow predispose these patients to bacteremia and embolic complications. Any unexplained fever should prompt blood cultures and inflammatory marker testing.

To enhance the clinical applicability, Table 3 summarizes the main preoperative, intraoperative, and postoperative risks associated with key CHD in adults undergoing NC surgery. This structured overview aims to facilitate perioperative planning and risk stratification.

### 3.5. Comorbidities in ACHD and Their Impact on Non-Cardiac Surgery

Comorbidities in ACHD become increasingly prevalent with age and the complexity of the cardiac defect [28,29,30]. The burden of co-existing conditions has been evaluated in NC surgeries using the Elixhauser Comorbidity Index, which was applied in the studies by Maxwell and Williamson to assess perioperative risk [4,5]. These comorbid conditions are strongly associated with an increased risk of mortality, emphasizing the need for comprehensive preoperative assessment. However, some comorbidities appear to be underrepresented, including frailty, immunodeficiency, and genetic syndromes.

The lasting pulmonary effects of CHD can create substantial perioperative challenges and amplify surgical risk. Research indicates that more than half (56%) of adults who had cardiac surgery in childhood develop restrictive lung disease, impacting ventilatory management and extubation strategies [31]. Furthermore, certain complex conditions, including heterotaxy syndrome, are associated with impaired ciliary function, which increases the likelihood of postoperative pneumonia and extended reliance on mechanical ventilation [32,33]. Renal dysfunction is a common comorbidity in ACHD, affecting approximately 40% of patients, with an even higher prevalence in those with Eisenmenger syndrome [34]. Renal function may be compromised postoperatively, requiring careful perioperative monitoring and individualized management to mitigate complications.

Frailty, traditionally linked to aging, is increasingly recognized in ACHD, affecting 5.8% of patients over 40 [35]. Frailty is a multidimensional, age-related condition marked by diminished physiological reserve, reduced adaptive capacity, and heightened vulnerability to stressors. It has been well established as a predictor of poor perioperative outcomes, including increased mortality, myocardial infarction, postoperative delirium, functional decline, and prolonged dependence on mobility aids or institutional care [36]. A multidisciplinary approach, including geriatric consultation and prehabilitation, can improve outcomes.

Emerging research indicates that CHD shares key characteristics with chronic inflammatory disorders, marked by persistent immune activation, dysregulation of lymphocyte subsets, and increased susceptibility to infections and immune dysfunction. Both humoral and cellular immune deficiencies, particularly antibody deficiencies, have been identified in CHD patients, often manifesting as recurrent infections, increased hospitalization rates, and a heightened risk of life-threatening complications, such as pneumonia and endocarditis [37]. Additionally, CHD patients are nearly twice as likely to develop immunodeficiency compared to the general population [37], with those experiencing cyanosis, heart failure, genetic syndromes (e.g., DiGeorge, Down, and Noonan syndromes), or post-surgical thymectomy being at the highest risk [38]. These patients exhibit chronic inflammation, T-cell senescence, altered immune regulation, and increased susceptibility to infections and autoimmune diseases, highlighting the need for comprehensive immune assessment and tailored perioperative management.

Some CHD patients are at increased risk of bleeding, such as cyanotic CHD or Fontan with liver disease (FALD) that progresses to fibrosis and cirrhosis, with a risk of coagulopathy and perioperative bleeding. Additionally, many ACHD patients require long-term anticoagulation, particularly those with mechanical valves and arrhythmias. The increasing use of direct oral anticoagulants (DOACs) has introduced new perioperative challenges, as these agents have different pharmacokinetics and reversal options compared to vitamin K antagonists. While DOACs are increasingly prescribed in ACHD patients, studies suggest higher bleeding risks compared to traditional anticoagulants [39]. The risks of withholding these agents and subsequent thromboembolic events must also be considered. Perioperative management of anticoagulation therapy should be tailored based on bleeding risk and procedural complexity [8].

## 4. Perioperative Risk Stratification and Referral Strategy in ACHD Patients

Perioperative risk stratification in ACHD patients must consider the severity of the congenital heart disease, the patient’s physiological status, and the inherent risk of the surgical procedure. Recently, the French Society of Anesthesiology (SFAR) proposed a three-tier risk classification based on anatomic and pathophysiologic classification defined by the 2018 American Heart Association guidelines and the risk related to the procedure [40,41]. Even if this classification needs to be validated, it can guide the appropriate referral of patients undergoing NC surgery and ensure optimal perioperative care.

Up to 74% of NC surgeries in ACHD patients take place in non-ACHD centers, primarily due to geographical constraints limiting access to specialized referral centers [42]. While elective NC surgery should ideally be conducted at a regional ACHD center, urgent or emergency procedures may necessitate management at non-ACHD hospitals. In such cases, it is crucial to consult the nearest ACHD expert center for perioperative guidance. Moreover, some low-risk elective surgeries may be safely performed in non-ACHD centers when the composite risk (related to the CHD severity and the procedure) is low. However, intermediate- and high-risk cases should be referred to ACHD centers to optimize outcomes.

Before elective surgery, a comprehensive review of the patient’s surgical history and current cardiac physiology is essential for accurate risk assessment. Suboptimal preoperative evaluation or optimization accounts for up to 40% of adverse events in ACHD patients undergoing non-cardiac surgery [10]. Ensuring a thorough perioperative assessment and multidisciplinary collaboration can significantly reduce complications and improve surgical outcomes.

## 5. Conclusions

ACHD patients undergoing NC surgery face significant perioperative challenges, necessitating a structured risk assessment approach. Comprehensive preoperative evaluation, including congenital heart lesion complexity, physiological status, and surgical risk, is essential to guide decision-making and referral strategies. While elective surgeries should ideally be performed at ACHD centers, urgent procedures may require management in non-specialized settings, highlighting the importance of expert consultation. Tailored perioperative strategies, including careful hemodynamic monitoring, anticoagulation management, and frailty assessment, can improve outcomes. Multidisciplinary collaboration remains central to optimizing perioperative care, reducing complications, and enhancing long-term prognosis in ACHD patients.

Despite increasing data, perioperative risk prediction in ACHD patients remains limited by the lack of validated, lesion-specific tools. Most available evidence comes from retrospective studies, often based on administrative data. Future research should focus on developing prospective, ACHD-specific risk models incorporating clinical, imaging, and biomarker data. Ongoing multicenter collaborations and registries will be essential to fill these gaps and guide individualized perioperative care.

## Figures and Tables

**Table 1:** Most common non-cardiac surgeries in adults with congenital heart disease across two study periods.

Procedure Type	Maxwell (2002–2009) [4]	Williamson (2010–2018) [5]	
General Surgery	35.1%	Colectomy	21.6%
		Gastrectomy	3.42%
		Hepatectomy	2.56%
		Abdominal Aortic Aneurysm	5.16%
		Esophagectomy	0.48%
Orthopedic Surgery	29.9%	Hip Replacement	48.7%
Thoracic Surgery	13.9%	Lung Resection	18.0%
Ear, Nose, Throat	2.3%	-	-
Gynecologic Surgery	7.7%	-	-
Neurosurgery	12.0%	-	-
Urologic Surgery	6.0%	-	-

**Table 2:** Surgical risk estimates according to the type of surgery or intervention from the ESC guidelines [8]. CAE, carotid artery endarterectomy; CAS, carotid artery stenting; VATS, video-assisted thoracoscopic surgery.

Low Surgical Risk (<1%)	Intermediate Surgical Risk (1–5%)	High Surgical Risk (>5%)
Breast surgery	Carotid asymptomatic (CEA or CAS)	Adrenal resection
Dental procedures	Carotid symptomatic (CEA)	Aortic and major vascular surgery
Thyroid surgery	Endovascular aortic aneurysm repair	Carotid symptomatic (CAS)
Eye surgery	Head or neck surgery	Duodenal-pancreatic surgery
Gynecological: minor	Intraperitoneal: splenectomy, hiatal hernia repair, cholecystectomy	Liver resection, bile duct surgery
Orthopedic minor (meniscectomy)	Intrathoracic: nonmajor	Esophagectomy
Reconstructive surgery	Neurological or orthopedic: major (hip and spine surgery)	Open lower limb revascularization for acute limb ischemia or amputation
Superficial surgery	Peripheral arterial angioplasty	Pneumonectomy (VATS or open surgery)
Urological minor: transurethral resection of the prostate	Renal transplants	Pulmonary or liver transplant
VATS minor lung resection	Urological or gynecological: major	Repair of perforated bowel
		Total cystectomy

**Table 3:** Perioperative risk profile by congenital heart disease type: ASD, atrial septal defect; ACHD; adult congenital heart disease; AKI, acute kidney injury; PFO, patent foramen ovale; CHD, congenital heart disease; D-TGA, dextro-transposition of the great arteries; ccTGA, congenitally corrected transposition of the great arteries; PVR, pulmonary vascular resistance.

CHD	Preoperative Risk	Intraoperative Risk	Postoperative Risk
ASD/PFO	Increased risk of perioperative ischemic stroke; echocardiographic screening may be needed	High stroke risk during major surgery due to paradoxical embolism	Elevated risk of stroke and mortality within 30 days
Fontan circulation	Severe hemodynamic vulnerability; cyanosis increases risk; frequent end-organ dysfunction	Hypoxia, hypotension, and arrhythmias common; anesthesia and mechanical ventilation reduce preload and cardiac output	Escalation of care, AKI, and heart failure in a small proportion of cases
Systemic right ventricle (D-TGA/ccTGA)	High risk of arrhythmias and systemic ventricular dysfunction	Bradycardia, bleeding, and failed extubation; systemic right ventricle may poorly tolerate anesthesia-induced changes, especially in severe dysfunction; limited outcome data in adults	Limited data; possible postoperative arrhythmia and heart failure
Cyanotic CHD/Eisenmenger syndrome	Fixed PVR, severe cyanosis, coagulopathy, and embolic risk; requires careful optimization	Profound hypotension and desaturation; highly sensitive to anesthetic induction and hemodynamic shifts; require use of vasopressor High risk of paradoxical embolism	High risk of decompensation and death
ACHD-related comorbidities	Frailty, restrictive lung disease, renal impairment, immune dysfunction, and genetic syndromes are prevalent	Increased ventilatory challenges; higher bleeding risk from anticoagulation or liver dysfunction	Risk of delirium, infections, thromboembolic or bleeding events, and prolonged recovery

## Abbreviations

The following abbreviations are used in this manuscript:

ACHD	Adult congenital heart disease
ACS-NSQIP	American College of Surgeons National Quality Improvement Program
ASD	Atrial septal defect
CAE,	Carotid artery endarterectomy
CAS	Carotid artery stenting
CCHD	Cyanotic congenital heart disease
CHD	Congenital heart disease
NC	Non-cardiac
RCRI	Revised Cardiac Risk Index
SVR	Systemic vascular resistance
TGA	Transposition of the great arteries
VATS	Video-assisted thoracoscopic surgery
